# Supplementary material for: Epidemiology of snake envenomation from Mato Grosso do Sul, Brazil
Source: PLoS Negl Trop Dis. 2021 Sep 8;15(9):e0009737. doi: 10.1371/journal.pntd.0009737 (PMC8452014; doi:10.1371/journal.pntd.0009737)
Supplement: S1 Table — (DOCX) [file pntd.0009737.s001.docx]

# Epidemiology of snake envenomation from Mato Grosso do Sul, Brazil

Karoline Ceron¹*, Cássia Vieira¹, Priscila Santos Carvalho¹^,^², Juan Fernando Cuestas Carrillo¹, Jaqueline Alonso¹, Diego José Santana¹

^1^Mapinguari - Laboratório de Sistemática e Biogeografia de Anfíbios e Répteis, Instituto de Biociências, Universidade Federal de Mato Grosso do Sul, Cidade Universitária, 79002-970, Campo Grande, Mato Grosso do Sul, Brazil.

² Instituto de Biociências, Letras e Ciências Exatas, Universidade Estadual Paulista (UNESP), Rua Cristóvão Colombo 2265, 15054-000, São José do Rio Preto, SP, Brazil.

* Corresponding author

E-mail: adenomera@gmail.com.

**SUPPORTING INFORMATION**

Supplementary Material S1

S1 Table. The total and the mean number of snake envenomation per 100,000 population for municipalities between 2008-2009 and 2011-2017 in Mato Grosso do Sul state (Brazil).

| **ID** | **Municipality** | **2008** | **2009** | **2011** | **2012** | **2013** | **2014** | **2015** | **2016** | **2017** | **Mean** |
| --- | --- | --- | --- | --- | --- | --- | --- | --- | --- | --- | --- |
| 1 | Figueirão | 147.58 | 116.18 | - | 101.87 | 33.37 | 133.11 | 199.20 | 33.11 | 66.07 | 103.81 |
| 2 | Paranhos | 69.95 | 112.52 | 111.87 | 102.58 | 45.72 | 97.66 | 81.52 | 102.38 | 79.41 | 89.29 |
| 3 | Tacuru | 189.81 | 146.54 | 87.12 | 57.46 | 46.40 | 36.67 | 36.25 | 107.53 | 35.45 | 82.58 |
| 4 | Rio Negro | 58.90 | 118.72 | 139.83 | 120.55 | 40.09 | 60.62 | 81.47 | 41.06 | 41.37 | 78.07 |
| 5 | Bodoquena | 83.32 | 47.64 | 125.69 | 75.68 | 50.13 | 37.79 | 101.29 | 63.62 | 115.09 | 77.81 |
| 6 | Laguna Carapã | 83.49 | 49.74 | 137.09 | 15.07 | 58.39 | 43.26 | 71.26 | 112.72 | 111.47 | 75.83 |
| 7 | Bandeirantes | 16.54 | 83.32 | 135.87 | 90.40 | 44.46 | 88.77 | 59.08 | 103.20 | 58.87 | 75.61 |
| 8 | Aquidauana | 95.10 | 90.29 | 63.35 | 71.83 | 55.52 | 61.70 | 65.73 | 71.85 | 77.92 | 72.59 |
| 9 | Pedro Gomes | 81.94 | 46.85 | 50.48 | 63.44 | 75.87 | 101.91 | 51.32 | 77.54 | 78.09 | 69.72 |
| 10 | Caracol | 76.09 | 56.39 | 73.26 | 72.46 | 87.73 | 69.34 | 51.39 | 67.73 | 66.98 | 69.04 |
| 11 | Jateí | 76.67 | - | 49.90 | - | - | - | - | - | - | 63.28 |
| 12 | Camapuã | 73.74 | 73.90 | 110.16 | 95.53 | 36.31 | 50.91 | 50.98 | 21.88 | 43.81 | 61.91 |
| 13 | Miranda | 85.07 | 64.42 | 62.03 | 84.66 | 74.99 | 55.78 | 70.10 | 32.95 | 25.43 | 61.71 |
| 14 | Porto Murtinho | 71.74 | 103.05 | 51.51 | 63.76 | 43.31 | 42.84 | 90.83 | 35.96 | 47.40 | 61.16 |
| 15 | Antônio João | 116.04 | 45.80 | 72.55 | 60.03 | 35.11 | 46.45 | 80.65 | 68.62 | 22.71 | 60.88 |
| 16 | Alcinópolis | 45.05 | 22.15 | 21.56 | 106.29 | 40.96 | 100.79 | 39.70 | 97.77 | 57.83 | 59.12 |
| 17 | Inocência | 53.04 | 79.99 | 65.33 | 65.45 | 12.97 | 26.02 | 39.14 | - | 78.76 | 52.59 |
| 18 | Iguatemi | 79.55 | 32.85 | 66.79 | 73.02 | 25.93 | 38.62 | 38.37 | 31.77 | 63.14 | 50.00 |
| 19 | Rio Verde de Mato Grosso | 36.59 | 52.04 | 26.39 | 36.83 | 36.17 | 66.99 | 41.11 | 56.37 | 86.87 | 48.82 |
| 20 | Caarapó | 46.93 | 59.08 | 34.41 | 26.38 | 47.18 | 64.28 | 73.85 | 38.11 | 44.38 | 48.29 |
| 21 | Nioaque | 51.12 | 38.23 | 48.82 | 49.00 | 41.73 | 62.92 | 42.16 | 35.31 | 35.48 | 44.97 |
| 22 | Taquarussu | 62.54 | 63.19 | 28.41 | - | 28.01 | 56.02 | 28.01 | - | - | 44.36 |
| 23 | Sidrolândia | 42.89 | 58.17 | 57.39 | 51.17 | 31.23 | 28.16 | 31.16 | 50.97 | 47.64 | 44.31 |
| 24 | Amambai | 57.97 | 42.87 | 28.46 | 50.67 | 38.16 | 35.00 | 53.21 | 42.07 | 44.20 | 43.62 |
| 25 | Juti | 18.12 | 17.96 | 66.99 | 49.68 | 32.05 | 31.64 | 46.88 | 77.21 | 45.78 | 42.92 |
| 26 | Coxim | 45.78 | 21.26 | 46.50 | 46.36 | 39.46 | 24.21 | 57.33 | 63.19 | 39.01 | 42.57 |
| 27 | Rio Brilhante | 51.03 | 39.42 | 35.17 | 40.78 | 44.96 | 29.34 | 48.88 | 47.93 | 27.67 | 40.58 |
| 28 | Nova Alvorada do Sul | 88.50 | 71.02 | 17.72 | 34.46 | 43.24 | 20.96 | 25.44 | 19.79 | 28.89 | 38.89 |
| 29 | Corguinho | - | - | 60.48 | 19.79 | - | - | 36.28 | - | - | 38.85 |
| 30 | Bonito | 33.73 | 39.20 | 40.43 | 50.04 | 43.70 | 28.81 | 28.51 | 51.72 | 32.58 | 38.75 |
| 31 | Costa Rica | 10.59 | 41.61 | 24.97 | 38.70 | 42.47 | 41.72 | 20.50 | 20.17 | 99.21 | 37.77 |
| 32 | Jardim | 37.43 | 45.50 | 20.42 | 36.56 | 19.86 | 43.43 | 58.89 | 23.42 | 42.71 | 36.47 |
| 33 | Ribas do Rio Pardo | 25.28 | 39.85 | 42.31 | 46.33 | 31.21 | 39.47 | 38.85 | 25.50 | 37.69 | 36.28 |
| 34 | Dois Irmãos do Buriti | 20.79 | 62.22 | 47.88 | 38.03 | - | 45.96 | 27.36 | 18.10 | 26.95 | 35.91 |
| 35 | Vicentina | 51.84 | - | - | 50.68 | - | - | - | 16.57 | 16.55 | 33.91 |
| 36 | Água Clara | 22.02 | 36.03 | 54.47 | 52.40 | 50.22 | 14.07 | 13.82 | 6.79 | 53.36 | 33.69 |
| 37 | Aral Moreira | 20.98 | 51.66 | 9.60 | 85.04 | 9.08 | 17.84 | 35.09 | 17.26 | 50.97 | 33.06 |
| 38 | Guia Lopes da Laguna | - | 28.83 | 9.70 | 39.01 | - | - | - | 69.56 | 10.01 | 31.42 |
| 39 | São Gabriel do Oeste | 18.85 | 41.57 | 8.84 | 43.45 | 16.64 | 32.63 | 24.02 | 35.37 | 42.47 | 29.32 |
| 40 | Brasilândia | 24.01 | 31.90 | 42.31 | 67.76 | 33.49 | - | 8.40 | 8.41 | 16.86 | 29.14 |
| 41 | Coronel Sapucaia | 20.81 | 61.78 | 70.62 | 7.02 | 13.69 | 6.80 | 6.75 | 20.11 | 53.28 | 28.98 |
| 42 | Sete Quedas | 63.86 | 63.90 | 27.86 | 9.30 | 27.58 | - | 9.23 | 9.25 | 18.54 | 28.69 |
| 43 | Chapadão do Sul | 35.75 | 46.26 | 24.68 | 35.05 | 14.11 | 18.22 | 17.68 | 21.47 | 20.89 | 26.01 |
| 44 | Novo Horizonte do Sul | 39.42 | 20.28 | 20.72 | - | 21.83 | 22.51 | - | 23.96 | 24.75 | 24.78 |
| 45 | Bela Vista | 21.22 | 25.29 | 30.06 | 25.65 | 29.30 | 29.16 | 16.59 | 33.03 | 8.22 | 24.28 |
| 46 | Corumbá | 36.29 | 35.19 | 17.25 | 18.11 | 17.70 | 26.85 | 35.89 | 10.98 | 18.20 | 24.05 |
| 47 | Sonora | 30.79 | 22.50 | 19.69 | 19.19 | 12.09 | 17.63 | 22.88 | 50.16 | 16.31 | 23.47 |
| 48 | Selvíria | 15.13 | 45.07 | - | - | 15.56 | - | 15.49 | - | - | 22.81 |
| 49 | Paranaíba | 37.39 | 24.84 | 22.32 | 12.36 | 21.83 | 21.76 | 16.87 | 21.62 | 21.55 | 22.28 |
| 50 | Fátima do Sul | 20.70 | 10.35 | 10.51 | 31.54 | 5.19 | 31.19 | 15.61 | 31.25 | 41.71 | 22.00 |
| 51 | Cassilândia | 27.85 | 32.29 | 9.51 | 9.48 | 18.61 | 41.75 | 13.87 | 13.83 | 27.59 | 21.64 |
| 52 | Angélica | 40.21 | 40.19 | 21.45 | - | 10.17 | 10.01 | 9.85 | 9.70 | - | 20.23 |
| 53 | Maracaju | 37.58 | 12.31 | 15.68 | 25.58 | 9.73 | 21.38 | 18.57 | 20.44 | 17.78 | 19.89 |
| 54 | Terenos | - | 39.28 | 22.77 | 27.82 | 10.56 | 10.29 | 10.04 | 4.91 | 28.77 | 19.30 |
| 55 | Rochedo | - | - | - | - | - | 19.21 | - | - | - | 19.21 |
| 56 | Aparecida do Taboado | 9.79 | 24.24 | 22.10 | 17.46 | 33.71 | 4.15 | 24.58 | 16.16 | 19.94 | 19.13 |
| 57 | Itaquiraí | 57.32 | 11.36 | 21.24 | 10.50 | 10.17 | 5.02 | 4.96 | 9.80 | 33.92 | 18.25 |
| 58 | Itaporã | 15.64 | 15.47 | - | - | 17.99 | 13.29 | 13.10 | 34.45 | 16.99 | 18.13 |
| 59 | Eldorado | 8.13 | 48.31 | 25.55 | 8.48 | 16.63 | 8.28 | - | - | 8.18 | 17.65 |
| 60 | Deodápolis | 60.42 | 8.62 | 8.20 | 8.16 | 15.97 | 15.89 | 15.81 | 15.73 | 7.83 | 17.40 |
| 61 | Rita do Pardo | 13.54 | - | 27.37 | - | 13.28 | 13.19 | - | - | - | 16.85 |
| 62 | Ponta Porã | 20.11 | 25.02 | 22.73 | 14.92 | 8.36 | 18.77 | 11.53 | 11.34 | 16.74 | 16.61 |
| 63 | Jaraguari | - | - | - | - | - | - | - | 14.41 | - | 14.41 |
| 64 | Mundo Novo | 6.08 | 24.23 | 5.83 | - | 22.65 | 11.25 | 22.37 | 16.67 | 5.52 | 14.33 |
| 65 | Bataguassu | - | 10.21 | 14.91 | 9.81 | 4.73 | 23.30 | 9.18 | - | 26.80 | 14.13 |
| 66 | Naviraí | 4.46 | 6.58 | 14.84 | 22.96 | 14.05 | 15.78 | 25.23 | 9.55 | 9.40 | 13.65 |
| 67 | Nova Andradina | 4.45 | 15.25 | 12.94 | 27.59 | 10.18 | 2.00 | 13.75 | 17.39 | 19.00 | 13.62 |
| 68 | Ivinhema | 18.92 | 23.73 | 4.47 | 8.91 | 13.14 | 17.48 | 8.72 | 17.41 | 8.69 | 13.50 |
| 69 | Dourados | 15.46 | 11.07 | 10.58 | 14.45 | 16.39 | 10.47 | 8.93 | 15.78 | 16.51 | 13.29 |
| 70 | Anaurilândia | 11.58 | 23.00 | 11.72 | 11.66 | - | - | 11.31 | 11.25 | 11.20 | 13.10 |
| 71 | Batayporã | 9.20 | 9.19 | - | 18.21 | 8.95 | 8.94 | - | - | - | 10.90 |
| 72 | Glória de Dourados | 10.09 | - | - | - | - | 9.99 | 10.01 | - | - | 10.03 |
| 73 | Três Lagoas | 4.52 | 15.64 | 10.62 | 8.55 | 4.56 | 8.06 | 10.56 | 10.38 | 11.07 | 9.33 |
| 74 | Campo Grande | 7.36 | 6.22 | 7.66 | 8.82 | 4.69 | 7.24 | 6.44 | 7.06 | 7.32 | 6.98 |
| 75 | Ladário | 5.41 | - | - | - | 4.74 | 4.65 | - | 4.50 | - | 4.82 |
| 76 | Anastácio | - | - | - | - | 4.08 | - | - | - | - | 4.08 |
