# Supplementary material for: Epidemiology of snake envenomation from Mato Grosso do Sul, Brazil
Source: PLoS Negl Trop Dis. 2021 Sep 8;15(9):e0009737. doi: 10.1371/journal.pntd.0009737 (PMC8452014; doi:10.1371/journal.pntd.0009737)
Supplement: Attachment — Submitted filename: Answers to reviwers comments.docx [file pntd.0009737.s002.docx]

**March 19, 2021**

**To Dr. Arunasalam Pathmeswaran**

**Associate Editor**

**PLOS Neglected Tropical Diseases**

**Response Letter**

Thank you for the opportunity to submit a revised version of our manuscript. Please find below the responses to the review of PNTD-D-21-00473: **“Epidemiology of snake envenomation from Mato Grosso do Sul, Brazil”** by Ceron et al. First of all, we greatly appreciate the careful and constructive comments made by the editor and referees during the review process and I would like to thank all of you for helping to improve our paper. In general, we improve the grammar throughout all the manuscript, updated the figures according to referees’ suggestions, and modified the predictors' variables of the GLM analysis (population density, biome, and municipality areas) to avoid correlated variables. All comments were individually and carefully addressed along the revision process and almost all points raised were incorporated in the revised version of our manuscript. Below we provide a point-to-point response clarifying only the main concerns.

**#Reviewer 1**

General

Thank you for the opportunity of reading and reviewing this very important piece of evidence about Snakebite Envenoming in Mato Grosso Do Sul, Brazil.

However, it leaves the reader and probably some snakebite specialists with a little sense that it is unfinished, and not completely focused A/ on epidemiology (incidence rates, comparisons, stratifications, environmental risk factors) B/ or demographic/clinical risk factors outcomes (severity criteria, snake genus,... ).

Your paper would benefit by adding :

1) a table with demographics (age, sex, profession, area, hospital...) of victims

2) a table with main outcomes : severity, deaths, compared to non-severe -> Odds ratios of dying? of being severe ? (by Month/Season? by Year ? by hospital (qlty of care)?

3) a map with estimated incidence rates per 100k pop/yr using the official census total population (it's ok if you explain the possible bias due to not having community data).

Looking forward to seeing the next version !

Many thanks for this opportunity and best wishes.

**Response: Thank you very much for yours comments. To improve the manuscript as suggested we added a map of envenomations by 100k pop and also a table with envenomations by 100k pop/year, to turn our results comparable among municipalities. Unfortunately, the Brazilian data about snake envenomations does not provide clinical information about victims or even so the numbers of deaths by month. So, with this manuscript we intend to explore the first trends of snake envenomations at Mato Grosso do Sul, once there are no reports about this. We tried to report all data available that can be important to build an epidemiologic scenario.**

1. Some limitations to address goals: using this design it is difficult to infer any incidence rates (per 100,000 / yr ) as the N of SBE cases who did not consult a health facility are not recorded. This type of study often under-estimates the true incidence.

**Response: Thank you for your comment. This is the main issue involving snakebites manuscripts around the world, mainly in countries in developing (as Brazil). The main objective of this work is to give the first estimate of snakebites accidents in the region, and since it, find the gaps in this type of study in the region and improve them.**

2. Population: Denominator is not stated - who is the population addressed (total district/region population or beyond)?

**Response: We added a map showing the number of cases by 100k pop, which we hope, will fill this gap.**

3. Number of snakebites per snake genus: how was this be assessed in this retrospecive study (at initial assessment, or post-hoc by retrospecive study authors)?- please explain a little more.

**Response: Envenomations by animals are considered by the medical team as moderate or severe, and they are the ones who identify which species caused the accident and the type of venomous, according to victims’ symptoms. We added a sentence in methods explaining data obtention by SINAN (lines 85-94). Thus, the genus is assessed at initial assessment by health professionals.**

4. "Elapid accidents were treated as severe..." -> do you mean considered as neurotoxic ? indeed elapids bites can also be dry non-envenomed bites - not all are severe as the quantity of venom injected can be small. A bit unclear.

**Response: According to FUNASA, 2001 in the guide “Manual for the diagnosis and treatment of accidents involving poisonous animals” all cases of coral accidents** **with clinical manifestations should be considered as potentially serious. We modified this sentence to be cleaner, now it reads (lines 96-99): “Elapidic accidents with clinical manifestations were treated as severe, lethetic accidents were treated as moderate to severe depending on the symptoms, and botropic and crotalics varied among mild, moderate, and severe [19].”**

5. "Lethetic"? I do not undertand this word. Do you mean by Lachesis?

**Response: Lachetic accidents. We corrected this word (line 97).**

6. Methods lines 114 to 122 : you present Results in the Methods chapter. I think this is more appropriate in the Results chapter.

**Response: We removed the results and maintained only the part of the carried-out analysis (lines 114-118).**

Results

7. We usually present the total N of snakebite victims, rather than "snakebites events/accidents". This is because of the possible duplicates. Did any patient consult twice the same year or separate years (ID number identical)?

**Response: Unfortunately, the database does not provide this information. It only reports the number of accidents per site/month. Because of this, we opted to maintain the term events.**

8. Lines 132-133 , 152-155, etc...: I am not a statistician, but I think that you do not need to present so many details (F value, x2 value and df value): in most studies P-value is sufficient for these simple statistics (You already explained their choice in methods).

**Response: Thank you for your comment, however, we choose to give all statistics results to give them clear to the reader. These values give a notion of sample size and strength of the analysis.**

9. Line 114 (results in methods) compared with Figure 1 : you say "January (n = 6090, February (n =535)"... I am surprised by this number for January - is it 6090 or 609 ??

**Response: We removed this part of methods according question #6. The right number was 609.**

10. -155: "Differences in snake genera" : It is not clear how this genus was determined : visually by the victim? killed and brought to the clinic/hospital ? extrapolated by the initial clinician ? or by the authors retrospectively?

**Response: Envenomations by animals are considered by the medical team as moderate or severe, and they are the ones who identify which species caused the accident and the type of venomous, according to victims’ symptoms. We added a sentence in methods explaining data obtention by SINAN (lines 85-94).**

11. - 163: Deaths : it would be useful to know a bit more about these 15 deaths (0.27%). The CFR seems low. Did they occur in children, women? Early deaths (first 24hours) or later ? Syndrome associated with mortality? (rather than species which can be often mistaken).

**Response: Unfortunately, this is also not reported by the database. It does not provide a timeline by cases; thus, we cannot relate the cases with mortality or aggravates.**

12. Recovery : Most people (84.88%) recovered successfully... What about the 15% who did not recover successfully? - amputations? sequelae? long-term disability? Lost to follow-up % ?

**Response: Thank you for your comment. We inserted in this sentence the information about the 15% remaining. Now it reads (lines 162-165): “In contrast, most of the people (84.88%, n = 4.227) who suffered snakebites recovered successfully, one person died by other causes not related to the snakebite (0.01%) and, 826 people (14.83%) did not inform about case evolution”**

13. - 169 : grave : I think you mean severe (severe in table 3).

**Response: Word corrected (line 166).**

14. - How was the categorisation between mild, mod, severe - which criteria ? (Brazilian guidelines? WHO? Other?)

**Response: Envenomations by animals are considered by the medical team as moderate or severe, and they are the ones who identify which species caused the accident and the type of venomous, according to victims’ symptoms. We added a sentence in methods explaining data obtention by SINAN (lines 85-94).**

15. - 174-178: There is no comparison between number of cases and population (incidence per 100k pop/y) of each geographical area. This would be very important (essential ) to use this type of study, for any epidemiological comparison with studies from other parts of the world.

**Response: We added a map showing the number of cases by 100k pop and a table of envenomations by 100k pop/y.**

16. - Fig3 is interesting showing N of cases per area and showing a superior number in the Pantanal biome areas; BUT for epidemiologists this figure should be based on numbers per 100,000 inhabitants - this would allow comparisons and would possibly show different colour gradients based depending on pop density.

**Response: We added a map showing the number of cases by 100k pop**

17. -> You could use your total population (2,449,024 in 2010) or rural population (15% only?) as a denominator. Does the percnt of rural pop (< or <15%) vary among the districts in your map. This is important as it can bias your interpretation. Therefore the total population may be more reliable.

**Response: We thank you for your comment. We changed the data used in analysis and now we teste against snake envenomations: population density, municipality area and biome.**

18. - Fig 4 (b): I am not sure I understand what you mean by "rural population" on the x-axis ? Is this the total population minus the urban population ? I think it would be clearer on the map (Like map Fig 3).

**Response: We thank you for your comment. We changed the data used in analysis and now we teste against snake envenomations: population density, municipality area and biome.**

Conclusions

19. Yes but not entirely : you do not really report incidence rates, nor comparative incidence rates. N-cases cannot really be compared if there is no denominator (total population).

**Response: We added a map indicating the incidence by 100.000 population to turn it comparable.**

20. - Lines 205-207- "these data may not reflect the real number of snakebites as some accidents were not reported because they happened in remote rural areas where victims do not have the access to health care." -> I agree with your statement BUT: would it be possible to estimate (from other studies) the proportion of victims (incl women) who only consult basic health posts or traditional healers in remote areas? In other countries and all continents , this is our major issue leading to under-estimation (Costa Rica, India, Nepal, Sri Lanka, Cameroon, etc.). It would be interesting to conduct a random multicluster cross-sectional study in the same area to assess this difference in incidence.

**Response: We thank you for your comment. Yes, here in Brazil this is also true in relation to snakebites. The majority of studies that relate to this issue are concentrated in Northeast and North Brazil (e.g., https://doi.org/10.1016/j.jep.2014.12.020 and https://doi.org/10.1016/j.toxicon.2019.10.238), despite the Mato Grosso do Sul have a huge indigenous population. We think this goal cannot be addressed in this paper because it requires a major review of literature, but it should be an interesting objective to another manuscript.**

21. - Your hypothesis on agricultural growth seems interesting but it is not supported by data from the Results chapter (not really a conclusion).

**Response: Yes, it is only a hypothesis related to the higher number of reports. We added other explanation to this, that could be more reasonable, now it reads (lines 205-215): “The highest number of notifications observed in 2017 could be related to the major use of the notification platform by the municipalities in the face of modernization of prefectures, allowing internet access to the majority of municipalities resulting in a higher number of accidents reports. Despite this, it can be related to the growth in the agricultural sector in that year [26], because agricultural activity itself is a risk factor for the occurrence of snakebites worldwide [27]. In 2017, agriculture grew 9.2% in comparison with 2016, with production breaking records in several crops, especially soy (23.9%) and corn (62.9%) [28].”**

22. - I have doubts about this conclusion: "There was no significant difference between the number of accidents and the victim’s sex even though 75.90% occurred in men and only 24.08% in women." - If you take the total N F and M in the total population (50 & 50) this would give you a very significant difference in a simple 2x2 table (X-square , p-value<0.00001).

**Response: We thank you for your comment. We now performed the test using the total values of females' and males’ accidents, and not the accidents by year. Now, the result was positive. We modified the methods (lines 109-111) and the results (lines 130-132).**

23. - Same question on age groups: 20-29 and 30-39. P-value comparing age groups? e.g. severe vs mild-mod? In many studies children are significantly more severe, and mortality is higher in children (also in women in some areas in Asia).

**Response: Thank you for this comment. No doubt, this can relation can be very informative; however, we do not have this information (severity) based on ages.**

Minor comments

24. Annually, 1,8 to 2,7 million snakebites 37 occur worldwide and 81 thousand to 138 thousand deaths --> Actually WHO says : 4.5 to 5.4 million bites / 1.8 to 2.7 million snakebite ENVENOMING cases (SBE)... 81-138 thousand deaths.. https://www.who.int/health-topics/snakebite#tab=tab_1 : "WHO...available data show 4.5–5.4 million people get bitten by snakes annually. Of this, 1.8–2.7 million develop clinical illness and 81 000 to 138 000 die from complications."

**Response: Thank you for this comment. We modified this sentence to include the correct information. Now it reads (lines 36-40): “Annually, 4,5 to 5,4 million snakebites occur worldwide, of this, 1,8 to 2,7 million develop clinical illness and 81 thousand to 138 thousand die as a consequence of those accidents. Estimates support that 400 thousand people suffer consequences such as restricted mobility, amputation, blindness, and post-traumatic stress”**

25. - Line 46 : hind limbs (usually for animals) -> lower limbs (for humans)

**Response: Word changed.**

26. - Line 61: epidemiologic profile of the snakebites : Do you mean present clinical profiles ... and relate them to epidemiological factors-variables?

**Response: No, only the epidemiologic panorama, which includes the distribution of accidents by year, genre, age, snake genera, and region. We think our data does not include clinical profile, once we did not present data per victim.**

27. - Lines 72 to 85: not sure these details on biodiversity threats are relevant for the analysis.

**Response: Thank you for this comment. Since one of our objectives is to compare the accidents by biome, we think it is useful to contextualize each biome to the reader. We reduce this paragraph by giving only essential information to the manuscript (lines 73-79).**

28. - Abstract, last line: "The number of snakebite accidents had a positive relationship with the size of the municipality and the population size living in rural areas." it is not clear if this is an association with population size" or with the rural vs urban environment?

**Response: We modified this sentence. Now it reads (lines 27-28): “To the Mato Grosso do Sul state, the total number of snake envenomations had a positive relationship with the size of the municipality.”**

**#Reviewer 2**

General

This manuscript needs some attention but study has validity. I would not use the terms "snakebite" "accident" or "snake accident" in this manuscript. This terminology is inaccurate and not attractive for those in the field. Correct terminology is snake envenomation and this needs to be replaced throughout the manuscript. Many people are bitten by snakes and we need to accurate on the terminology used. I also suggest that using the term "poisoning" is also not accurate when discussing envenomation. Line 96: "poisoning levels" what does that mean? I would also refer to "non-poisonous" snake as "non-venomous" as this is accurate and replace throughout manuscript. Remember, venom is a poison, but a poison is typically referred to when a toxin is ingested, inhaled, or absorbed through the skin. Venom is injected directly into the body via "bite or sting" from an organism.

**Response: Thank you for this comment. As suggested, we change the terms snakebite, accident, and snake accident to snake** **envenomation throughout the manuscript. We also change the word poison and its derivates (non-poisonous) to non-****venomous.**

1. Study area: how many known venomous and non-venomous snake species exist?

**Response: We added a sentence indicating this. Now it reads (lines 74-76): “Mato Grosso do Sul is located in three different biomes, Cerrado, Pantanal, and Atlantic forest, harboring 10 species of venomous snakes and 103 species of non-venomous snakes”.**

2. Data collection: We need a better description of the national database where the data was collected. Who is collecting it? How is it entered? Who is identifying the snakes? What is the process at which a clinician would enter the data?

**Response: SINAN (Sistema de Informação de Agravos de Notificação) is a platform for the collection and processing of data on notification diseases at the national level, which are notified by health professionals. Thus, it contributes to the identification of the epidemiological reality of a given geographical area and allows an analysis of the morbidity profile. The data are obtained by standardized notification form according to each condition of national interest. Accidents by venomous animals are considered by the medical team as moderate or severe, and they are the ones who identify which species caused the accident and the type of venomous. All these information was added in the material and methods section to make it clearer.**

3. We need to distinguish between "snakebite" and "snake envenomation" throughout the manuscript. I have hard time accepting that "poisoning levels" were determined by species and there is no significant clinical data available in this database aside from mortality?

**Response: We modified the term snakebite to snake envenomation throughout all manuscript. In Brazil, there is a clinical guide to classify envenomation by snakes. As example, according to FUNASA, 2001 in the guide “Manual for the diagnosis and treatment of accidents involving poisonous animals” all cases of coral accidents with clinical manifestations should be considered as potentially severe. The envenomation by other species must be classified according the victims’ symptoms. The database used in this manuscript is from Sistema de Informação de Agravos e de Notificações (SINAN) from Ministério da Saúde do Brasil. It platform only provide general numbers of envenomation with no information about clinical profile or other individual information.**

4. One major question I have is "snakebite" mean "snake envenomation" for data entry.

**Response: Yes. As mentioned above, the platform informs the numbers of snake envenomation that occurred in Brazil. This clinical information is validated by medical in health units under the observation of symptoms and victims relate.**

Results

5. : Table 3: how did we determine severity of the envenomation? What does ignored mean? Does that mean snake was not identified?

**Response: In Brazil, there is a clinical guide to classify envenomation by snakes. As example, according to FUNASA, 2001 in the guide “Manual for the diagnosis and treatment of accidents involving poisonous animals” all cases of coral accidents with clinical manifestations should be considered as potentially severe. The envenomation by other species must be classified according the victims’ symptoms by the medical.**

6. Is there any clinical data available to report? Appears limited

**Response: No, only the epidemiologic panorama, which includes the distribution of accidents by year, gender, age, snake genera, and region.**

7. Do we have information on the circumstances of the "snakebites" like occupation and other factors placing increased for a snake encounter?

**Response: Unfortunately, the database does not provide this information.**

8. "non-poisonous" snakes bites were 3.02% and I would consider that low for such a large area. Is this because the initial thought was it was a venomous snake and it was later deemed non-venomous encounter? To only have 168 non-venomous snake encounters over such a large period indicates that the individual entering the data must have thought it was venomous but it was later determined not to be a venomous encounter. We need clarification.

**Response: To count in the database, probably, the victim showed some symptoms after the snakebite. However, these symptoms may not show progress and the health professional may indicate as a non-venomous encounter. As we added in methods, SINAN (Sistema de Informação de Agravos de Notificação) is a platform for the collection and processing of data on notification diseases at the national level, which are notified by health professionals. The data are obtained by standardized notification form according to each condition of national interest. Accidents by venomous animals are considered by the medical team as moderate or severe, and they are the ones who identify which species caused the accident and the type of venomous.**

9. Among the 15 who died, do we have any more information on the cases? Delay in antivenom or health care?

**Response: Unfortunately, the database does not provide this information.**

10. I would remove the work "accidents" from the manuscript and use envenomation.

**Response: We removed the word accidents and replace them with envenomation.**

11. I would consider a figure of the common genera that are found in this region associated with snake envenomation.

**Response: We provide a figure illustrating the common species found in the state.**

Conclusions

12. No limitations are present and there are several. Including data collection and data entry system, which is largely the biggest limitation. We need more discussion on the process of data entry. Is every suspected snake envenomation required to be entered into the system? Who determined the snake envenomation and severity of illness?

**Response: SINAN (Sistema de Informação de Agravos de Notificação) is a platform for the collection and processing of data on notification diseases at the national level, which are notified by health professionals. Thus, it contributes to the identification of the epidemiological reality of a given geographical area and allows an analysis of the morbidity profile. The data are obtained by standardized notification form according to each condition of national interest. Accidents by venomous animals are considered by the medical team as moderate or severe, and they are the ones who identify which species caused the accident and the type of venomous. We added a sentence in methods explaining data obtention by SINAN (lines 85-94). Also, we improve the discussion about the limitation of data and how notifications can be underestimated.**

We thank you for your consideration and time, and we look forward to hearing from you.

Yours sincerely,

Karoline Ceron

Corresponding author
