# Supplementary material for: Epidemiology of snake envenomation from Mato Grosso do Sul, Brazil
Source: PLoS Negl Trop Dis. 2021 Sep 8;15(9):e0009737. doi: 10.1371/journal.pntd.0009737 (PMC8452014; doi:10.1371/journal.pntd.0009737)
Supplement: Attachment — Submitted filename: Answers to reviewers comments.docx [file pntd.0009737.s003.docx]

**July 9, 2021**

**To Dr. Arunasalam Pathmeswaran**

**Associate Editor**

**PLOS Neglected Tropical Diseases**

**Response Letter**

Thank you for the opportunity to submit a revised version of our manuscript. Please find below the responses to the review of PNTD-D-21-00473: **“Epidemiology of snake envenomation from Mato Grosso do Sul, Brazil”** by Ceron et al. Below we provide a point-to-point response clarifying only the main concerns.

**#Reviewer 1**

General

1) Looking at your responses to points 3 and 4, I think it is still unlear how you classified elapid bites (eg by Micrurus/coral) when you look at table 3, in which you classify most bites by Micrurus as mild (22) and moderate (4) and only 7 as severe. This does not fit your answer about coral snakebites considered as "severe" (Point 4 below): elapid accidents were treated as severe... it contradicts the classification of Micrurus bites into mild-moderate-severe. I think it's better that you stick to the initial clinical classification as you have no means to verify retrospectively. So I would remove your extrapolation (also the corrected one) saying that  "Elapidic accidents with clinical manifestations were treated as severe" (lines 96-99).

**Response: Thank you for this comment. As mentioned in our text, there is a guide that orient the classification of snakebites according to the snake, FUNASA, 2001 in the guide “Manual for the diagnosis and treatment of accidents involving poisonous animals”. However, the classification is filled by the medical professional at the moment of the examination. Thus, we will follow your recommendation and remove the lines 96-99 (now 128-130), and we will give it clear that the classification is attributed by the health professional (lines 120-123): Envenomations by animals are considered by the medical team (from mild to severe). The snake genus that caused the accident and the type of venomous is identified at the initial assessment by the health professionals, according to victims’ symptoms.**

2) I do not understand how reliable this table is as it does not seem to be associated with final outcomes (death, surgery, amputations, disability, hospitalisation time).

**Response: Thank you for the comment. By this table, we would like to characterize the envenomations, and how it is distributed according to the snake genus. We also included a sentence of this in the discussion (lines 282-283): …** **Despite this, the majority of cases related to the genus *Bothrops* were classified as mild, according to the symptoms.**

3) **This does not correspond to your answers :
   3. Number of snakebites per snake genus: how was this be assessed in this retrospecive study (at initial assessment, or post-hoc by retrospecive study authors)?- please explain a little more.
   **Response: The genus is assessed** **at initial assessment by health professionals. They characterize the genus by examining the symptoms of victims: Now it reads (lines 120-123): Envenomations by animals are considered by the medical team (from mild to severe).** **The snake genus that caused the accident and the type of venomous is identified at the initial assessment by the health professionals, according to victims’ symptoms.**
4) Envenomations by animals are considered by the medical team as moderate or severe, and they are the ones who identify which species caused the accident and the type of venomous, according to victims’ symptoms. We added a sentence in methods explaining data obtention by SINAN (lines 85-94). "Elapid accidents were treated as severe..." -> do you mean considered as neurotoxic ? indeed elapids bites can also be dry non-envenomed bites - not all are severe as the quantity of venom injected can be small. A bit unclear.

**Response: Thank you for this comment. As mentioned in our response #1, there is a guide that orient the classification of snakebites according to the snake, FUNASA, 2001. However, the classification is filled by the medical professional at the moment of the examination. Thus, we will follow your recommendation and remove the lines 96-99 (now 128-130), and we will give it clear that the classification is attributed by the health professional (lines 120-123): Envenomations by animals are considered by the medical team (from mild to severe). The snake genus that caused the accident and the type of venomous is identified at the initial assessment by the health professionals, according to victims’ symptoms.**

5) Another point is your answer about having a map with N cases per 100k? I do not find it : the map still shows absolute number of cases,  but it does not show  Cases/100k pop./year  -> It would be great to modify the red map and the colour scale of reds to reflect the real relative incidence rates/100k pop.,

**Response: The map was attached to the submission platform. We also added below for your convenience**

6) In the limitations you should talk about the usual under-estimation due to cases and deaths ocurring outside any medical facility. You would need a random cluster household survey - the next step ?

**Response: Thank you. In the discussion section, there is a paragraph elucidating the study limitations (lines 235-240): Additionally, these data may not reflect the real number of snake envenomations as some envenomations accidents were not reported because they happened in remote rural areas where victims do not have the access to health care [24], as related in others studies carried out in Brazil [e.g., 7,10,22]. Besides that, many patients prefer to use alternative medicine rather than a modern treatment provided by a health center. Unfortunately, this incidence is poorly addressed in Latin America, but it probably plays a significant role in underestimating the incidence and possibly severity of envenomations [25].**

**We also added a paragraph of conclusion with a statement for the need for more detailed studies in the region (lines 364-369): Together, our data highlight the general scenario of snake envenomations in West Brazil and their factor risk involved (working-age males, in bigger municipalities with an economy based on agricultural activity with low population density). In addition, we highlight the urge for more detailed studies involving snake envenomations in West Brazil, clarifying the epidemiologic pattern in focal populations and also the clinic evolution of cases.**

**#Reviewer 2**

General

7) Need to include in the methods section the explanation on severity scale as it pertains to the FUNASA, 2001. For those outside of Brazil, this method of grading clinical snake envenomation may be different. A statement, like provided in the response letter should be added to lines 127-130 and also referenced.

**Response: Thank you for this comment. We modified this sentence according to reviewer #1 suggestion: As mentioned in our text, there is a guide that orient the classification of snakebites according to the snake, FUNASA, 2001 in the guide “Manual for the diagnosis and treatment of accidents involving poisonous animals”. However, the classification is filled by the medical professional at the moment of the examination. Thus, we will follow your recommendation and remove the lines 96-99 (now 128-130), and we will give it clear that the classification is attributed by the health professional (lines 120-123): Envenomations by animals are considered by the medical team (from mild to severe). The snake genus that caused the accident and the type of venomous is identified at the initial assessment by the health professionals, according to victims’ symptoms.**

8) In the response letter the authors have acknowledged limitations and this needs to be added to the discussion section. The analysis is descriptive and based-off data entered by health professionals into the SINAN database. Clinical data is not provided and details of the envenomation are lacking. A paragraph of limitations should be added and would add more rigor to their study. It also is a chance to introduce a statement for the need for more detailed study on snake envenomation in this region of Brazil.

**Response: In the discussion section there is a paragraph elucidating the study limitations (lines 235-240): Additionally, these data may not reflect the real number of snake envenomations as some envenomations accidents were not reported because they happened in remote rural areas where victims do not have the access to health care [24], as related in others studies carried out in Brazil [e.g., 7,10,22]. Besides that, many patients prefer to use alternative medicine rather than a modern treatment provided by a health center. Unfortunately, this incidence is poorly addressed in Latin America, but it probably plays a significant role in underestimating the incidence and possibly severity of envenomations [25].**

**We also added a paragraph of conclusion with a statement for the need for more detailed studies in the region (lines 364-369): Together, our data highlight the general scenario of snake envenomations in West Brazil and their factor risk involved (working-age males, in bigger municipalities with economy based on agricultural activity with low population density). In addition, we highlight the urge for more detailed studies involving snake envenomations in West Brazil, clarifying the epidemiologic pattern in focal populations and also the clinic evolution of cases.**

9) - the term accidents is scattered throughout the manuscript. In my opinion, "accidents" should be replaced with envenomation. It appears that some studies coming out of Brazil are using the term "accident" for envenomation. The more common terminology should be used and envenomation is more exact.

**Response: Thank you for this comment. We revised the entire manuscript and changed “accidents” by “envenomations”.**

10) - limitations are not clearly discussed in the discussion section. Others from Brazil have been published using data in the SINAN database and this should also be stated.

**Response: Thank you. We improve the discussion of limitations in lines 235-240: Additionally, these data may not reflect the real number of snake envenomations as some envenomations accidents were not reported because they happened in remote rural areas where victims do not have the access to health care [24], as related in others studies carried out in Brazil [e.g., 7,10,22]. Besides that, many patients prefer to use alternative medicine rather than a modern treatment provided by a health center. Unfortunately, this incidence is poorly addressed in Latin America, but it probably plays a significant role in underestimating the incidence and possibly severity of envenomations [25].**

**We also added a paragraph of conclusion with a statement for the need for more detailed studies in the region (lines 364-369): Together, our data highlight the general scenario of snake envenomations in West Brazil and their factor risk involved (working-age males, in bigger municipalities with an economy based on agricultural activity with low population density). In addition, we highlight the urge for more detailed studies involving snake envenomations in West Brazil, clarifying the epidemiologic pattern in focal populations and also the clinic evolution of cases.**

We thank you for your consideration and time, and we look forward to hearing from you.

Yours sincerely,

Karoline Ceron

Corresponding author
